# Dental Hygiene

**Published:** 1884-05

**Authors:** S. B. Palmer


### DENTAL HYGIENE; THE BEST VARIETIES OF FOOD TO DEVELOP AND SUSTAIN TOOTH STRUCTURE.

BY S. B. PALMER, M. D. S.

READ BEFORE THE FIFTH DISTRICT DENTAL SOCIETY OF THE STATE OF NEW YORK, AT ITS ANNUAL MEETING AT UTICA, APRIL 8 AND 9, 1884.

Dental hygiene is a system of principles or rules for the preservation of the dental organs. In one short paper we can only mention a few of the general rules to be observed. Good inheritance, good health, and proper care of the teeth are necessary for sound dentures. We must not consume time by going back of our subject. To start even with it, the most of our directions must be communicated to the children, through their mothers, or those having the care of them.

The object in writing is to impress upon you, particularly the younger members, the importance of instructing your patients in the care of their teeth.

Please excuse me for addressing you in popular terms, as though you were patients rather than dentists. Whoever does his patient good must instruct in terms to be understood, and offer suggestions that can be practiced. It is well known that mothers who understand how to live, and what to eat, can do much for the child during the period of gestation and lactation. The services of a dentist are seldom required for children under four years of age. At whatever age it may be, none of the temporary teeth should be allowed to decay so as to expose the pulp, if possible to prevent it. The foundation for good teeth really antedates the birth of the child. Previous to birth, the lime elements, which compose the larger portion of teeth, are gathered from the nourishment taken by the mother. Unless her food is rich in phosphates, or bone-making material, not only will the child's teeth be soft, but the teeth of the mother will be drawn upon to furnish the needed supply, and they, too, become soft and sensitive, as every dentist knows from observation.

Thus the mission of a dentist in the cause of humanity extends beyond the limits of filling teeth and furnishing substitutes for those lost. Our legislators have wisely passed a bill for teaching hygiene in schools, and I trust dental hygiene will not be overlooked in the text-books to be introduced. We should teach patients, so far as practicable, that teeth are organized bodies, composed of animal and mineral matter. The latter is mainly lime in various forms, which makes up nearly seventy per cent. of the tooth substance. The animal portion is cartilage; it contains life, circulation, sensibility, etc. Through this medium the tooth receives more or less of the mineral elements upon which teeth depend for hardness and durability. During the period from conception to the leaving off of milk aliment, the growth of bones and teeth is greatly influenced by the quantity and quality of the phosphates furnished by the mother, and the milk diet. Following we give a few condensed suggestions relative to food and diet, collated from the expressed views of the best writers upon this subject.

Analysis of the human body shows a combination of thirteen simple elements, all of which have been extracted from elements contained in the food, and, through the circulation of the blood, are conveyed to the various portions to be built up and sustained.

Though lime-water and phosphates are administered with beneficial results, we look to food as the natural method of furnishing materials for the growth of the bones and teeth. Milk contains all the elements necessary for the growth and development during infancy. It is important that this nourishment should not be robbed of any of its lime, if good teeth are expected. Nearly all food adapted to man's subsistence contains the necessary elements for the growth of the bones and teeth. Unfortunately, advanced civilization renders it fashionable to sift from our cereals the outer coatings of grain, especially of wheat, which alone contains all the calcium or lime elements found in the grain, and so necessary for the growth of teeth. Modern improvements in milling render it unnecessary to allow coarse bran to enter into pastry, in order to get the benefit of the Graham flour, which in former years was so unpalatable. Though sold under the name of Graham flour, and possessed of all the elements of former times, it is objectionable only in color. When the real merits of Graham flour, oatmeal, and other articles of food which contain the natural phosphates, come to be appreciated, sensible people will greatly increase the demand for such preparations.

Thus far we have considered the supply for tooth formation as furnished through natural channels, and as contained in food. Many families are so situated that they must take such food as they find upon the table, and, from neglect or careless habits of eating, find the system so deranged as not to assimilate the proper elements, even though contained in their food. Medical science has discovered a remedy for this evil in "soluble phosphate of lime," "syrup of lacto-phosphate of lime," etc. This condition often occurs during gestation and lactation. Do not fail to urge upon your patients the use of lime-water, or carbonate of soda in water, with which to rinse the mouth after each meal, and after eating acid fruits. These simple and harmless remedies often give great relief where teeth become sensitive at the necks or margin of the gums. It is the duty of every dentist to insist upon proper care of children's teeth. First, teach the mother or nurse; second, the patient when old enough to comprehend the instruction. In many cases I have utterly refused to operate for neglected or neglectful children, without a promise to aid in the duty of cleanliness. A dentist who regards his reputation cannot afford to fill teeth in neglected mouths. Failure of his best work will be inevitable, and blame will surely follow. Reliable investigation proves that decay in teeth, which is not from a lack of sufficient lime-salts, is the result of acids, not directly upon the whole surface of the teeth, but between teeth, in fissures or seams, where starchy food or meat fibres lodge, till decomposition or fermentation takes place, which requires only five or six hours at the temperature of the mouth. Thus the importance of cleansing teeth after eating, and before retiring, may be readily seen. During sleep the fluids of the mouth remain inactive, and decomposition sets in, acid fermentation is the result, and cavities are formed or enlarged. Therefore, urge the use of a tooth-pick and floss silk to aid in removing lodgments of food. The frequent use of lemons and acid fruits greatly injures the enamel and bone of the teeth, not so much by the formation of cavities, as by decalcification of the tooth surface, and at the neck of the teeth where the enamel is thin.

Most patients can understand what is meant by "teeth-edge," which is sensitiveness caused by acids. Tell them that the acid has commenced to dissolve the lime, and thus to expose the sensitive animal portion ; tell them that long continuance of such exposure will dissolve away the lime and leave the teeth in a condition too sensitive for use. The lime-water above mentioned is the proper remedy.

Let us recapitulate. To produce and maintain good, sound teeth, the work must commence with the mother. The child, too, must be nourished with food containing all the elements found in teeth.

Food must not be allowed to remain around and between the teeth till it ferments and becomes an acid. Avoid extreme use of lemons and strong acids.

Rinse the mouth after eating acid fruit. See that teeth are properly cleansed during any illness. See that tartar or lime does not form around the necks of teeth, as it inflames the gums and eventually causes teeth to loosen and fall out.

Urge cleanliness and frequent watching of the teeth under your care. So will you benefit your patients and honor your profession.

